
JOURNAL INFORMATION
==============================
NLM Title Abbreviation: ScientificWorldJournal
Journal ID: ScientificWorldJournal
NLM Journal ID: 101131163
Journal ID: TSWJ

The Scientific World Journal

ISSN: 2356-6140
EISSN: 1537-744X
Publisher: Wiley

ARTICLE INFORMATION
==============================
PMCID: PMC6009570
PMID: 29973854
DOI: 10.1100/tsw.2002.293
Article ID: 785389
Article version: 1
Subjects: Editorial

The ICPEP-2 Meeting in India: Biodiversity to the Rescue!

Manning William J. *
University of Massachusetts, Amherst, MA, USA
*William J. Manning: aamanning@mindspring.com
Publication date: 2002
Electronic publication date: 2002 May 3
Volume: 2
First page: 1196
Last page: 1197
Received 2002 Apr 3; Revised 2002 Apr 5; Accepted 2002 May 3
Copyright: Copyright © 2002 William J. Manning.
Copyright year: 2002
License: This is an open access article distributed under the Creative Commons Attribution License, which permits unrestricted use, distribution, and reproduction in any medium, provided the original work is properly cited.
License URL: https://creativecommons.org/licenses/by/3.0/

Keywords: bioindicators, biomonitors, phytoremediation, passive samplers

==============================
**Editorial**
*TheScientificWorldJOURNAL* (2002) 2, 1196–1197
ISSN 1537-744X; DOI 10.1100/tsw.2002.293

### The ICPEP-2 Meeting in India: Biodiversity to the Rescue!

William J. Manning
*University of Massachusetts, Amherst, MA*

E-mail: aamanning@mindspring.com

*Received April 3, 2002; Accepted April 5, 2002; Published May 3, 2002*

**KEY WORDS:** bioindicators, biomonitors, phytoremediation, passive samplers

**DOMAINS:** terrestrial environmental toxicology, environmental monitoring, environmental management and policy, biomarkers, risk and impact assessment, bioremediation and bioavailability, environmental technology

I was pleased to be invited to participate in the Second International Conference on Plants and Environmental Pollution (ICPEP-2), held at the National Botanical Research Institute (NBRI) in Lucknow, India, from 4–9 February 2002. I was 1 of 250 delegates, mostly from India, but also from China, Nepal, Thailand, Sri Lanka, Bangladesh, U.S., and several European countries. It was most encouraging that many of the delegates were enthusiastic young scientists who were able to interact and build networks so vital to future collaborative science in this region.

The conference began with a ceremony that was unusual for foreign visitors like me. Invited guests took turns lighting different wicks on a large oil-filled “lamp of learning.” This was accompanied by several readings about the value of science and the inherent value of every living thing. It established a very respectful and appropriate climate for the conference. I was quite impressed. I think an opening ceremony like this would be very appropriate for meetings in the Northern Hemisphere.

Professor M.S. Swaminathan, the father of the Green Revolution in India, gave the opening lecture and set the theme for the meeting. His lecture was entitled: “Biodiversity: An Effective Safety Net Against Environmental Pollution.” His message was that species diversity can be used to protect humans from environmental pollutants.

Where species diversity is high, plant and animals species can be selected that can be used as bioindicators or biomonitors for a wide variety of pollutants. This allows early detection of adverse effects and evaluation of the effectiveness of laws that are designed to reduce pollution inputs.

Species diversity also allows us to select lines of plants that are capable of removing toxicants from soils and water. These plants can be used to bioremediate contaminated areas.

©2002 with author.

Phytoremediation is an important and rapidly expanding area of science that holds great promise in cleaning up hazardous areas.

Many of the papers and posters at the meeting reflected Professor Swaminathan's theme. There were a number of reports of the effective use of algae and cynaobacteria as effective bioindicators of water quality. Macrophyte (higher plants) diversity was also advocated as a sensitive bioindicator of lake and reservoir quality. Terrestrial plants and lichens are being used to monitor air pollution around industrial sites and in cities. Criteria included seed germination and seedling growth and plant growth and reproduction.

Because of its low cost and lack of technical problems, phytoremediation is quite popular in this region. Considerable effort is being devoted to identifying local plant species that can be used to remediate pollutants such as pharmaceutical wastes, arsenic, fly ash, and metals. This type of technology is fairly low cost, does not require extensive equipment, and is appropriate for India, Pakistan, Bangladesh, and other Asian nations.

Physical and chemical monitoring of environmental pollutants in this region is a problem, due to costs and lack of appropriate equipment and expertise. In terms of air pollutants, which are extensive in India, an alternative technology may be quite useful. Low-cost, simple passive samplers have been developed to detect cumulative concentrations of air pollutants, such as sulfur dioxide, nitrogen oxides, and ozone. These can be used to assess concentrations in air that may adversely affect humans and plants. There is an unfortunate trend, however, to assume that exposure to these pollutants equals a response. In the case of plants, this may not be the case, as environmental conditions must be ideal for pollutant uptake and effects to occur. This leads to misleading data and possible overestimation of the effects of air pollutants on plants. My invited paper addressed this tendency and pointed out the necessity of relating monitored concentrations of air pollutants to plant effects to give biological credibility to the air pollutant monitoring data.

The NBRI has an extensive collection of books and journals that relate to the environmental sciences and environmental pollution. In honor of ICPEP-2, they arranged a special exhibit of publications from their library. I was asked to officially open this exhibition, by cutting the ribbon across the door to the library. This was a new experience to me and I felt a special kinship with the British Royal Family, whose members perform such activities with great regularity.

My journey to India was long and tiring, but I am glad that I went, as I met some very interesting and friendly people from India and surrounding countries. I especially enjoyed meeting young scientists and students. The conference was very important for them and as a result it also became important for me.

---

**This article should be referenced as follows:**

Manning, W.J. (2002) The ICPEP-2 meeting in India: biodiversity to the rescue! *TheScientificWorldJOURNAL* **2**, 1196–1197.

---
